# Single-Cell Transcriptomics: Technical Advances, Applications and Challenges in Cancer Drug Discovery

**DOI:** 10.2174/0115680096372729250715050452

**Published:** 2025-07-30

**Authors:** Yueying Yang, Lingyu Meng, Teng Zhang, Jianjun Tan

**Affiliations:** 1 Department of Biomedical Engineering, School of Chemistry and Life Science, Beijing University of Technology, Beijing 100124, China;; 2 Beijing International Science and Technology Cooperation Base for Intelligent Physiological Measurement and Clinical Transformation, Beijing 100124, China

**Keywords:** Single-cell RNA sequencing, cancer drug discovery, target identification, heterogeneity, tumor microenvironment, drug resistance, therapeutic

## Abstract

With advancements in technology, single-cell RNA sequencing has emerged as a powerful tool in cancer drug discovery. This technique enables the construction of gene expression profiles at the individual cell level, offering detailed insights into cellular heterogeneity and molecular pathways involved in tumor development. It enables researchers to gain a deeper understanding of tumor heterogeneity. Researchers can study cell subpopulations and gene expression patterns. This understanding helps in identifying potential drug targets. Additionally, it aids in predicting therapeutic responses. This high-resolution gene expression analysis provides a new perspective and opportunity for cancer drug development, which is expected to accelerate the discovery and development process of new anti-cancer drugs. This article provides a comprehensive overview of the basic processes and developmental trajectory of single-cell RNA sequencing technology, with a particular emphasis on its applications in various aspects of cancer drug discovery. It also addresses the challenges faced by single-cell RNA sequencing and potential future directions. This review aims to enhance readers’ understanding of single-cell sequencing, inspire new ideas for oncology drug development, and support the translation of clinical research into practice, ultimately enabling physicians to design more precise and personalized treatment strategies.

## 1 INTRODUCTION

The global cancer incidence and mortality are rapidly increasing. The International Agency for Research on Cancer predicts that the number of new cancer cases worldwide will increase by 77% by 2050 compared to 2022 [1]. Investments in oncology research and drug development have also increased, making cancer one of the largest therapeutic areas in the pharmaceutical industry. However, the development of cancer drugs is inefficient [2-4]. These inefficiencies are partly due to our limited understanding of oncology, particularly in terms of the disease mechanisms, actionable therapeutic targets, and heterogeneity of disease responses [5, 6]. Cancer is a highly heterogeneous disease composed of malignant cells and a complex tumor microenvironment. The tumor microenvironment (TME) refers to the environment surrounding a tumor, comprising surrounding blood vessels, immune cells, fibroblasts, signaling molecules, and the extracellular matrix. TME plays a crucial role in promoting or inhibiting mechanisms such as tumorigenesis, invasion, and metastasis. During drug development, cell heterogeneity often leads to difficulties in identifying targets and drug resistance [7, 8].

Traditional batch sequencing methods, while providing overall average gene expression information, often mask intercellular heterogeneity. The advent of single-cell RNA sequencing (scRNA-seq) has enabled researchers to resolve the tumor microenvironment (TME) at single-cell resolution. This technology allows for the detailed investigation of cellular states, intercellular interactions, and regulatory mechanisms within tumor cells and their surrounding environment. As a result, scRNA-seq has opened new avenues for tumor diagnosis, treatment, and drug development, particularly in the fields of targeted therapy and immunotherapy [9-12].

In this review, we introduce scRNA-seq technology and focus on the integration of scRNA-seq with drug discovery pipelines, including the application of scRNA-seq technology in the understanding of tumor diseases, target discovery, drug screening, and studies of drug resistance mechanisms (Fig. **1**). Furthermore, we elucidate the challenges encountered in scRNA-seq and propose avenues for enhancement.

### 1.1 Methodology

This review was conducted by searching the literature through "Web of Science" and "PubMed". The search keywords include "single-cell RNA sequencing", "cancer drug discovery", "target identification", "tumor microenvironment", "drug resistance", *etc*. The data range is from 2014 to 2024. We screen and classify the literature through manual sorting and maintenance. Firstly, we have preserved both classic and latest articles. The overall framework of this paper was then determined by comparing and classifying the literature. Our paper consists of three parts:  an overview of single-cell RNA sequencing technology, the application of single-cell RNA sequencing technology in cancer drug discovery, and the challenges and prospects. Finally, each chapter of this paper was written through a detailed analysis of the literature.

## 2 OVERVIEW OF SINGLE-CELL RNA SEQUENCING TECHNOLOGY

### 2.1 Basic Workflow of Single-cell RNA Sequencing Technology

Single-cell technology is a method of sequencing analysis that allows for the examination of individual cells to reveal intercellular heterogeneity. These techniques include single-cell transcriptome sequencing [13], single-cell genome sequencing [14], and single-cell epigenomics [15]. Single-cell omics sequencing technologies aim to decode the potential information encoded in DNA, RNA, and proteins within the central dogma of molecular biology. ScRNA-seq involves the sequencing analysis of individual cells at the transcriptome level.

Initially, gene expression analysis was typically conducted at the population level, which lacked detailed information about individual cells. When using bulk RNA sequencing (bulk RNA-seq), the transcriptomic differences of each cell were masked by averaged information from multiple cells [16, 17]. However, by analyzing each cell at the cellular level, we can obtain information on heterogeneity, which enables more accurate cell clustering and the discovery of new cell types. This also provides a more effective method for studying differential gene expression and exploring cell lineage pathways, which can yield more precise information for drug development [18].

A typical single-cell RNA-sequencing workflow (Fig. **2**) consists of three key stages: sequencing, pre-treatment, and data analysis [19, 20]. Sequencing involves single-cell isolation, mRNA capture, reverse transcription, cDNA amplification, library construction, and sorting. Subsequent computational steps were performed after the sequences were obtained. The basic format of the raw sequencing data obtained from scRNA-seq includes the Fast Quality (FASTQ) and Illumina Base Call File (BCL) formats, which must be preprocessed for counting and cleaning before obtaining a count matrix that can be used for downstream data analysis. Pre-treatment includes alignment, quality control, normalization, data correction, feature selection, and dimensionality reduction. Downstream data analysis mainly involves cell clustering, annotation, subtype identification, difference analysis, enrichment analysis, communication analysis, survival analysis, and pseudotime analysis, and may also require data integration and batch correction. These steps have very important applications in cancer research. For example, the breast cancer microenvironment is highly heterogeneous and consists mainly of tumor cells, mesenchymal stromal cells, and immune cells. A variety of cells in the breast cancer microenvironment may be the source of heterogeneity. However, traditional transcriptome sequencing technology cannot address the heterogeneity of the microenvironment. Single-cell isolation has advanced cancer research by enabling the study of tumor heterogeneity at the single-cell level. For example, Nguyen *et al.* [21] mapped the single-cell transcriptome of the human mammary epithelium, providing valuable insights into cellular diversity and lineage differentiation. The study employed single-cell transcriptome sequencing to map the transcriptome of a total of 25,790 normal mammary epithelial cells originating from 7 females. Following clustering analysis, three classes of cells were defined: secretory Luminal cells, hormone-responsive Luminal cells, and basal cells. The differentiation pathways connecting these three classes of cells were reconstructed using a proposed temporal sequence analysis. This study systematically describes the characteristic profiles of mammary epithelial cells, contributing to a better understanding of the genealogical differentiation of breast cancer cells.

### 2.2 Development of Single-cell RNA Sequencing Technology

ScRNA-seq is a high-throughput sequencing technology that enables the determination of RNA within individual cells,

thereby revealing cellular heterogeneity and gene expression profiles. In 2009, Tang *et al.* [22] first proposed a method for scRNA-seq, addressing the issue of RNA amplification in single-cell samples, and successfully demonstrated the possibility of analyzing gene expression at the single-cell level. Followingly, researchers explored this technology and developed dozens of scRNA-seq methods [23], as listed in Table **S1** of the Supplementary Materials. These methods have enabled necessary improvements and modifications to single-cell isolation, capture, reverse transcription, cDNA amplification, library preparation, and sequencing, among others, to enhance yield and automation while reducing time, cost, and noise. With technological advancements, scRNA-seq technology has also evolved from single-omics to multi-omics and spatial omics (Fig. **1**).

Single-cell isolation is the first and most critical step in scRNA-seq, and it is often a major source of contamination, batch effects, and procedural variability. Therefore, efficient, reliable, and accurate single-cell capture is crucial for high-throughput, unbiased single-cell sequencing [24, 25]. Single-cell isolation methods can be categorized into three main types: (1) Early single-cell isolation methods, including limited serial dilution, manual microscopy, and laser capture microdissection (LCM) [26]. Limited serial dilution refers to a method in which a sample is gradually diluted to obtain only one or a small number of cells in a specific volume. Manual microscopy involves microscopic observation, the selection of cells with good morphology, and the aspiration of individual cells using a micropipette. Laser capture microdissection (LCM) involves visualizing tissue under a microscope to identify target cells. The operator delineates the desired region on the monitor, and a laser precisely cuts along the defined path to isolate and remove the selected cells. These methods are low-throughput, time-consuming, inefficient, and technically challenging. (2) Fluorescence-activated cell sorting (FACS) [27] and magnetic-activated cell sorting (MACS) [28] are two commonly used high-throughput techniques. FACS is a single-cell suspension that has been labeled with a fluorescent probe and deflected differently by a high-voltage electric field to achieve the separation of target cells from non-target cells. Magnetic-activated cell sorting (MACS) uses antibodies or ligands conjugated to magnetic microspheres (magnetic beads) to label and isolate specific cell populations. Under the action of the magnetic field, the labeled cells carrying the label precipitate at the magnetic poles, while the unlabeled cells stay in the supernatant. Thus, the separation of labeled cells from unlabeled cells is realized. However, these methods have certain limitations, including the expense of flow cytometers and low sorting throughput. (3) In the context of current throughput sequencing platforms, involving microfluidic-based single-cell manipulation methods have emerged as leading technologies for single-cell isolation in transcriptomic studies [29-31]. These methods significantly improve the scale, efficiency, and accuracy of the isolation process. This primarily involves three methods: microwell-based, droplet-based, and integrated fluidic circuits-based, which enable simultaneous processing and analysis of tens of thousands of single cells. Microfluidics utilizes the fundamental principles of fluid dynamics to separate single cells in nanoliter or picoliter volumes. Microfluidic devices are often referred to as microfluidic chips. Microwell-based methods involve adding a cell suspension to a gel microwell mold and using gravity to cause the cells to fall into the microwells, which usually hold only one cell per microwell. In the droplet-based approach, the cell suspension is first pumped into the microfluidic chip, where it is combined with a suspension of microbeads bearing molecular barcodes to form a laminar flow. Subsequently, the aqueous phase converges with the oil phase to generate individual microdroplets, each encapsulating a single cell along with a microbead. Integrated fluidic circuits-based method, which utilizes an advanced photolithographic integration process to create a reaction array of many nanoscale microtubes and nanoscale microbubbles etched into silica gel. Independent nanoscale microvalves control the flow of the solution in the Reaction Chamber of the array to achieve the separation of biological samples.

Reverse transcription (RT) is another crucial step, and the sensitivity of scRNA-seq largely depends on its efficiency. It is estimated that only 10%-20% of transcripts are reverse-transcribed, causing high-tech noise. Currently, scRNA-seq primarily relies on detecting read counts at the 3' or 5' ends of polyadenylated transcripts, which do not provide sufficient coverage for mRNA splicing. Third-generation sequencing (TGS) [32], also known as long-read sequencing, is a real-time molecular sequencing technology that overcomes this limitation by detecting full-length cDNA and RNA [33, 34]. It has achieved coverage of full-length transcripts from the 3’ end or the 5’ end, thereby reducing the coverage bias caused by incomplete reverse transcription. Examples include SCAN-seq [35], SCAN-seq2[36] and MARS-seq [37].

Due to the extremely low levels of mRNA in individual cells, cDNA must be amplified after reverse transcription to generate sufficient material for sequencing library preparation. Polymerase chain reaction (PCR)-based amplification can rapidly and efficiently amplify large amounts of cDNA in a short time, which is limited by the inherent characteristics of the exponential amplification process. CEL-Seq [38] and CEL-seq2[39] employ in vitro transcription (IVT)-based linear amplification, representing an alternative method for cDNA synthesis and amplification. IVT is primarily achieved through in vitro transcription synthesis, utilizing DNA as a template. The process primarily involves using DNA templates containing either a T7 (TAATACGACTCACTATAGGG) or SP6 (ATTTAGGTGACACTATAG) promoter sequence to synthesize mRNA that is complementary to one of the DNA strands. This is achieved using T7 or SP6 RNA polymerase and nucleotide triphosphate (NTP) substrates. A large quantity of mRNA can be rapidly synthesized, and its stability is enhanced by adding a 5′ cap structure and a 3′ poly-A tail. A key advantage of in vitro transcription (IVT) is its linear amplification, which reduces amplification bias and improves accuracy and reproducibility compared to PCR.

The use of cellular barcodes and unique molecular identifiers (UMIs) is crucial for the development of single-cell technology. Cellular barcodes are an efficient strategy for parallel processing, which was initially employed in the STRT-seq method [40] in 2011. The same single-cell mRNA receives a short oligonucleotide sequence, cell barcoding, during reverse transcription to distinguish the transcriptome of individual cells. Using unique barcode information, these transcripts can easily be attributed to their respective cells. This method significantly reduces costs and increases sequencing throughput. Followingly, in 2014, Islam *et al.* [41] introduced UMIs to identify each cDNA molecule within a cell. Similar to cellular barcodes, UMIs are random oligonucleotide sequences of 6-8 base pairs that can be integrated into each transcript within a cell during reverse transcription. Due to the large number of UMIs, each transcript was assigned a unique barcode, ensuring that all duplicate molecules generated by PCR amplification retained the original transcript's UMIs. By counting the unique barcodes, the original copy number of the transcript can be accurately quantified, thereby eliminating PCR amplification bias [42-45].

Recent developments have enabled the simultaneous sequencing of multiple omics at the single-cell level, allowing for a comprehensive analysis of cellular biological characteristics and obtaining richer information, which increases understanding of cellular states and functions, and provides more direct and detailed insights into gene regulation [46-58]. Techniques such as ECCITE-seq [59], scNMT-seq [60], and DOGMA-seq [61] now allow simultaneous measurement of different reads (*e.g.*, RNA expression, surface protein expression, cloning, DNA methylation, and chromatin accessibility) from the same single cell.

Spatial location information is crucial for determining the cell fate. Spatial transcriptomics captures both gene expression and the physical locations of cells within tissues. It can help researchers better distinguish the location of transcription [62] and further enhance scientists' understanding and interpretation of individual cells [63-70]. Currently, the main spatial transcriptomic methods can be categorized into three types: spatial barcoding-based, laser capture microdissection-based, and image-based methods, such as 10x Visium [71], FISSEQ [72], STRP-seq [73], and Stereo-seq [74].

Simultaneously, as the scale of data increases, data analysis methods are continually being improved, and various data analysis tools and algorithms (such as Seurat [75] and Scanpy [76]) have been developed to process and analyze high-dimensional single-cell transcriptomic data, thereby promoting the interpretation of results and the mining of biological significance. Batch effect processing methods can effectively integrate data. Seurat and Scanpy are scRNA-seq analysis platforms written in R and Python, respectively. This typically involves converting raw reads into cell-gene count matrices through sequence alignment, followed by analyses such as filtering, high-variable gene selection, dimensionality reduction, clustering, and differential expression analysis. Among other things, cells and genes are filtered to remove those with poor quality and those with the lowest expression. The data are then normalized to account for non-biological sources of variability, such as sequencing depth, technical noise, library size, and batch effects. Seurat provides various data preprocessing and standardization methods that can effectively reduce technical noise and improve data quality. Moreover, Seurat's cell type annotation function is relatively powerful. Scanpy has strong processing capabilities for large-scale datasets. Additionally, due to the use of different algorithms and parameter settings, various tools or versions often yield varying results, so applications must be selected according to the specific situation. To better manage data and facilitate data exchange, researchers have established various single-cell databases, such as Gene Expression Omnibus (GEO)[77], Human Cell Atlas (HCA)[78], and scRNASeqDb [79]. With an increasing volume of data, various machine learning and deep learning methods have been introduced into data analysis, leading to significant progress in areas such as data processing, cell classification, and trajectory inference.

Currently, most clinical transcriptomic data are generated using high-throughput RNA-seq or microarrays. To improve data utilization, methods for integrating data between bulk and single cells have been proposed [80, 81]. Furthermore, cell type deconvolution methods can estimate cell type composition based on gene signatures derived from single-cell RNA sequencing (scRNA-seq) data [82].

ScRNA-seq has broad application prospects in fields such as biological research, elucidation of disease mechanisms, drug development, and personalized medicine. With the advancement of technology and gradual reduction in costs, it is expected that more researchers will be able to utilize this powerful tool and achieve results in both basic research and clinical applications.

## 3 APPLICATION OF SINGLE-CELL RNA SEQUENCING TECHNOLOGY IN CANCER DRUG DISCOVERY

ScRNA-seq technology plays a crucial role in the discovery of drugs for tumors. Its greatest advantage is its ability to analyze at the single-cell level, revealing tumor heterogeneity that bulk RNA-seq cannot uncover. By analyzing cells at the single-cell level, researchers can identify different cell subtypes and their gene expression profiles. This information deepens our understanding of disease mechanisms. By comparing healthy and diseased tissues, researchers can identify differentially expressed genes that are specific to certain cell types or disease states, thereby contributing to the discovery of new therapeutic targets. Single-cell technology has also played a significant role in the elucidation of drug resistance mechanisms (Fig. **1**).

### 3.1 Single-cell RNA Sequencing Technology for Disease Understanding

It is necessary to study the mechanisms underlying tumor development. Compared to traditional batch sequencing, scRNA-seq enables the resolution of tumors at the single-cell level, thereby enhancing our understanding of tumors [83, 84]. Cancer cells arise from normal somatic cells because of DNA mutations that cause them to lose their normal regulatory mechanisms. They can grow continuously, invade neighboring tissues, metastasize to distant sites, and promote the growth of new blood vessels, from which they obtain nutrients and develop into complex heterogeneous tumor masses. The tumor microenvironment (TME) comprises multiple cell types, including tumor cells, as well as numerous non-malignant cells such as immune cells, epidermal cells, and fibroblasts, along with extracellular matrices and cytokines. Research has shown that the TME plays a critical role in tumor growth, invasion, and metastasis. Analyzing the TME helps elucidate the mechanisms of tumorigenesis, evolutionary patterns, and the causes of drug resistance during treatment [85, 86].

Using cell aggregation and annotation in single-cell sequencing analysis, researchers can comprehensively characterize the composition and states of different cell types in TME [87], map tumor cell profiles, and subsequently understand the roles and responses of different cell types in disease. This facilitates the identification of tumor treatment mediators in different cell types. For example, different immune cells play distinct roles in tumorigenesis, and the composition of immune cells in various tumors exhibits distinct characteristics. Therefore, in the study of tumorigenesis, treatment, and other mechanisms, the proportion of different immune cells can be analyzed through immune infiltration analysis, quantitative research on immune cells in different tumor types can be conducted, and tumor immune maps can be drawn. Professor Zhang’s team has revealed single-cell immune maps of liver cancer, colorectal cancer, and pan-cancer using single-cell sequencing technology, providing important insights into the complexity of the TME [88-92].

Single-cell analysis of healthy and tumor tissues, or at different tumor stages, can identify differentially expressed genes in distinct cells. Combined with enrichment analysis, this method can identify and refine cell subtypes, discover new tumor biomarkers, and significantly aid in diagnosis, treatment, and prognosis. Using biomarkers, patients can be stratified to identify populations that may respond more precisely to specific treatments. Prognostic models can be constructed by combining the survival analyses. Gliomas are the major malignant brain tumors. Zhao *et al.* [93] enhanced the understanding of the different cellular compositions of astrocytoma through the combined analysis of single-cell RNA (scRNA) and bulk RNA. They investigated various factors affecting the prognosis of astrocytoma, identified IGFBP7 as a potential biomarker, and developed a predictive model that classified patients into high- and low-risk groups based on IGFBP7 risk scores. Survival analysis showed that the high-risk group had a poorer prognosis, providing a new perspective for the treatment of gliomas.

The cells within a tumor are dynamically changing, and the differentiation process of each cell is asynchronous. Analyzing the trajectory of cell development is crucial for understanding the formation and development of tumors. Pseudotime analysis considers the process of cell differentiation as a timeline. Sort single cells along the trajectory based on the similarity of their expression patterns, and determine the position of each cell on the timeline. Therefore, pseudotime analysis is used to simulate the dynamic changes that occur in cells. Numerous studies have demonstrated that epithelial cells play a crucial role in the development and progression of esophageal squamous cell carcinoma. Yang *et al*. [94] used the R package “monocle2” to perform pseudotime trajectory analysis, investigating the dynamic changes in gene expression of epithelial cells during the development of esophageal cancer.

Multicellular organisms rely heavily on intercellular communication between different cell types to accomplish their desired functions. Specifically, in TME, intercellular communication plays a crucial role in cancer progression and affects responses to therapy. Single-cell communication analysis can help enrich our understanding of cancer development and metastasis mechanisms, providing insights into new treatment strategies [95].

### 3.2 Single-cell RNA Sequencing Technology for Drug Target Discovery

Currently, the primary methods of cancer treatment include surgical resection, radiation therapy, and medication. The earliest drug therapy, also known as chemotherapy, uses cytotoxic drugs to kill both cancer cells and healthy cells. Later, targeted therapy, which utilizes drugs that specifically target cancer cells without affecting healthy cells, emerged. Its main advantages include the ability to selectively target cancer cells, fewer side effects than non-targeted therapies, and higher efficiency in killing cancer cells.

The discovery of new drug targets is the starting point for targeted drug discovery in tumors. ScRNA-seq has distinct advantages over bulk RNA-seq and proteomics in the discovery and validation of drug targets. Bulk RNA-seq provides average data from all cells in a sample, and the expression of low-abundance genes is often averaged out. This can mask important cell-specific responses, making it difficult to identify certain targets. ScRNA-seq, on the other hand, is capable of capturing transcriptomic information at the single-cell level, and analysis reveals complex molecular networks and signaling pathways within individual cells. Researchers can identify tumor-specific molecular markers at the single-cell level, helping scientists discover new disease-related targets and validate their therapeutic potential. This accelerates the discovery and validation of new drug targets. Certain targets may not be detectable across the entire cell population but are significantly expressed in specific cell subsets. Thus, drug development strategies have shifted from targeting specific factors to targeting specific cell types. By comparing scRNA-seq data from healthy and diseased tissues, researchers can identify genes that are differentially expressed in specific cell types or disease states. These genes are likely to serve as effective therapeutic targets. Drugs directed at them can act more precisely on the relevant cell populations, reducing dependence on traditional chemotherapy or radiotherapy and thereby enhancing treatment efficacy and improving patient survival rates.

Cancer stem cells (CSCs) are a small population of tumor cells with the ability to self-renew and differentiate, and are capable of initiating tumor growth and metastasis. They play a crucial role in tumor drug resistance and recurrence. Therefore, signaling pathways involved in regulating the maintenance and survival of CSCs have emerged as promising targets for cancer therapy. Li *et al.* [96] used scRNA-seq to analyze the tumor immune microenvironment of primitive cancer, revealing significant differences in cell infiltration patterns between tumor and lymph node metastasis samples. The results showed that RAC2 and PTTG1 double-positive CSCs exhibited the highest stemness properties and were significantly enriched in the metastatic lymph nodes. The enrichment of stem-like CSCs in metastatic lymph nodes provides new therapeutic targeting opportunities. Although scRNA-seq can identify cellular heterogeneity within tumors, it has some inherent limitations that we acknowledge. These factors include potential sampling bias, variable sequencing depth, and the impact of data processing algorithms on the results. To enhance the reliability of research results, additional methods such as Western blotting and immunohistochemistry should be employed for validation. By analyzing the expression levels of biomarkers such as RAC2 and PTTG1 through scRNA-seq, different sample models can be established, and the reliability of the targets can be further verified through clinical validation. Yao *et al*. [97] used scRNA-seq to investigate CSCs in bladder cancer and identified DBI as a potential key gene and therapeutic target for quiescent CSCs. Furthermore, they confirmed that paracetamol may be a candidate for targeting CSCs, thereby improving our understanding of CSC-targeted treatment strategies.

Circulating tumor cells (CTCs) are tumor cells that detach from tumor tissue and enter the bloodstream. They play a crucial role in tumor metastasis, as they can spread through the blood and colonize distant organs, leading to the formation of new tumor foci [98]. CTCs interact with various components of the TME, including the interaction between CTCs and tumor-associated macrophages, which promotes local inflammation and enables cancer cells to evade the immune system, facilitating their attachment to and invasion of distant metastatic sites. Therefore, targeting and eliminating CTCs is a promising strategy for preventing metastasis and improving patient prognosis [99].

Cancer-associated fibroblasts (CAFs) are stromal cells that have heterogeneous phenotypes and functions. They are key components of the tumor microenvironment (TME) and represent potential targets for cancer treatment.

Due to the heterogeneity of CAFs, the impact of CAF subgroups on lung cancer progression remains unclear, which hinders the translational progress of targeted CAF therapies. Chen *et al.* [100] identified the POSTN+CAFs subgroup, which may promote the formation of fibrotic structures and participate in immune suppression. This study also found that this subgroup is associated with cancer progression and adverse clinical outcomes. This study provides new insights into the treatment of non-small cell lung cancer.

With growing research on the tumor microenvironment (TME), tumor immunotherapy has gained increasing recognition [101]. Tumor immunotherapy does not directly kill cancer cells. Instead, it induces a tumor-specific immune response in the body through active or passive means, thereby exerting its function in inhibiting and killing tumor cells. It has advantages such as high specificity and efficiency, as well as sparing the body from harmful treatments. In a study of lung cancer, Li *et al*. [102] revealed the heterogeneity of immune cells in the TME using scRNA-seq technology and identified key genes associated with tumor progression and treatment response. These genes may encode cell surface receptors, signaling molecules, or other proteins related to cancer, making them potential therapeutic targets and providing important evidence for optimizing targeted therapies and immunotherapies.

### 3.3 Single-cell RNA Sequencing Technology for Drug Screening

Single-cell RNA sequencing (scRNA-seq) has shown great potential for drug screening. It can be combined with high-throughput screening (HTS) methods to simultaneously analyze the expression profiles of various drug perturbations, assessing the impact of a large number of compounds on specific cell types or disease models [103-105]. Compared to traditional high-throughput screening (HTS) methods, scRNA-seq provides a more detailed view of cell-type-specific changes. This enables the separation of confounding factors such as the cell cycle, allowing for a more accurate assessment of drug effects.

By comparing gene expression changes in individual cells before and after different drug treatments, researchers can assess the specific effects of drugs on various cell subpopulations and screen for candidate drugs with optimal efficacy and minimal side effects [106]. This approach not only accelerates the drug development process but also increases the success rate of clinical trials and reduces research and development costs. Duan *et al.* [107] developed a high-throughput drug screening platform to screen perhexiline maleate from more than 6,000 compounds by scRNA-seq analysis, which showed that perhexiline maleate reduced SREBP2 expression levels and reversed KRAS mutant-induced upregulation of the cholesterol synthesis pathway, and is a potential drug for targeting KRAS.

Moreover, scRNA-seq technology can reveal off-target effects and heterogeneous responses to drugs across different cell subpopulations, providing important information for the selection and optimization of candidate drugs [108]. McFarland *et al*. [108] developed MIX-Seq, a method that identifies drug mechanisms of action by analyzing mixed cellular responses to chemical or genetic perturbations across 100 or more cancer cell lines using scRNA-seq data. This approach helps reduce uncertainty and risk during drug development, thereby enhancing drug safety and efficacy.

 Wang *et al.* [109] introduced the scRank tool and showed that scRank employs target perturbation of gene regulatory networks to infer drug-responsive cell types from untreated scRNA-seq data. When applied to adult neural tube cell tumors and major depression datasets, scRank identified drug-responsive cell types, which are consistent with the literature. In addition, scRank accurately revealed macrophage subpopulations responsive to tanshinone IIA and their potential targets in myocardial infarction, which were experimentally validated.

### 3.4 Single-cell RNA Sequencing Technology for Analyzing Drug Resistance Mechanisms

ScRNA-seq can accurately monitor drug response and disease progression in clinical trials. By analyzing individual cells, researchers can track changes in gene expression patterns associated with drug treatment responses or resistance [110]. By identifying and analyzing subpopulations of drug-resistant cells, physicians can develop targeted therapies that specifically address the characteristics of these cells and mitigate their impact on sensitive cells. Understanding the mechanisms of drug resistance in tumors can help develop personalized treatment plans that are tailored to a patient's specific tumor characteristics. Moreover, identifying resistant subpopulations can lead researchers to explore combination chemotherapy or immunotherapy to overcome resistance. For example, combining conventional chemotherapeutic agents with novel targeted drugs could enhance the therapeutic effect. Based on changes in drug-resistant cells, doctors can promptly adjust treatment regimens in response to cancer progression and the emergence of drug resistance, thereby greatly improving patient prognosis. By comparing scRNA-seq data from resistant and sensitive cell lines, researchers can identify gene expression changes associated with resistance, enhance the accuracy of drug sensitivity predictions, and subsequently develop novel drugs or therapeutic strategies that target resistance. This longitudinal monitoring provides early indicators of treatment efficacy or disease recurrence, enabling the timely adjustment of treatment strategies. For example, triple-negative breast cancer (TNBC) is a particularly aggressive breast tumor with a high mortality rate, primarily owing to the development of resistance to chemotherapy. Researchers [111] utilized bulk RNA-seq and single-cell RNA-seq (scRNA-seq) to investigate the transcriptional landscape before and after the emergence of resistance to the chemotherapeutic drugs paclitaxel and doxorubicin, revealing significant transcriptional heterogeneity within TNBC cell populations. They identified rare cell subpopulations associated with resistance-related genes and found that epithelial-mesenchymal transition (EMT) is a common mechanism of resistance within these cell subpopulations. This plays a crucial role in understanding tumor heterogeneity and in developing more effective, personalized treatment strategies to overcome chemotherapy resistance in TNBC. Immune checkpoint inhibitors (ICIs) are a tumor immunotherapy approved by the US Food and Drug Administration (FDA). ICIs, as a class of drugs, exert anti-tumor effects by activating the body's immune response. Researchers have found that various tumor cells can inhibit T cell function by targeting immune checkpoints, thereby allowing tumor cells to evade immune surveillance. For example, tumor-associated macrophages (TAMs) bind to programmed death receptor-1 (PD-1) on the surface of T cells through programmed death ligand-1 (PD-L1), inhibiting anti-tumor immune responses. These ligands and receptor proteins facilitate tumor progression through intercellular communication, significantly impacting the effectiveness of treatment and intervention.

Therefore, analyzing intercellular communication in the tumor microenvironment can aid in cancer diagnosis, guide targeted therapy, and predict disease progression. Inhibitors such as Ipilimumab, Atezolizumab, and Tremelimumab have been developed to target immune checkpoints, including cytotoxic T lymphocyte-associated protein 4 (CTLA-4), programmed death protein-1 (PD-1), and its ligand, PD-L1. ICIs have played a crucial role in melanoma treatment [112-114]. Peng *et al*. developed a deep learning model that utilizes scRNA-seq data to screen ligand-receptor interactions for inferring intercellular communication. This model utilizes the gene co-expression pattern of ligands and receptors to analyze intercellular communication in human melanoma by comprehensively scoring the thresholds and products of gene expression in scRNA-seq data. The results indicate that the communication strength between cancer-associated fibroblasts, macrophages, and melanoma cells is very high. This may be related to the expression of immune checkpoints, providing a basis for further analysis of tumor immunotherapy. However, only approximately 30% of the patients respond to immunotherapy. Researchers analyzed the dynamic changes in T cells of patients receiving PD-1 inhibitor treatment using scRNA-seq. They found that the characteristic changes in T cell lineage before and after treatment were closely related to the clinical response of patients, especially the significant increase in tumor-associated antigen-specific T cells after treatment. Therefore, analyzing gene expression patterns in patient samples helps identify molecular features associated with drug responses or disease progression, guide treatment choices, and optimize patient prognosis [115, 116].

### 3.5 The Other Applications of Single-cell RNA Sequencing Technology in Drug Discovery

ScRNA-seq technology can be used to analyze intercellular heterogeneity in various diseases. Therefore, He *et al.* proposed a single-cell-guided pipeline to aid in the reproduction of drugs [117], which is based on scRNA-seq to address intercellular heterogeneity in each patient's body, define drug scores to recommend drugs, and is a promising tool for drug reuse.

Although methods for predicting drug response using scRNA-seq data have been proposed, an integrated tool from scRNA-seq analysis for drug discovery is needed. Xie *et al.* developed a bioinformatics workflow, scDrug [118], which includes a one-step pipeline for generating cell aggregates for scRNA-seq data and two methods for predicting drug therapy. Through improvement, scDrug+ [119] was developed, which uses single-cell transcriptomics and molecular structures to predict drug responses and enhance predictive ability.

Clustered Regularly Interspaced Short Palindromic Repeats (CRISPR) technology plays a crucial role in drug screening [120, 121]. Single-cell CRISPR screening techniques, such as Perturb-seq [122], combine CRISPR screening with scRNA-seq or single-cell multi-omics, allowing for decoding the effects of individual perturbations on gene expression, their interactions, or their cell state dependencies, as well as prioritizing the cell types most sensitive to CRISPR-mediated perturbations at the single-cell level, observing drug responses, and improving drug target discovery efficiency. Liu *et al.* [123] used Perturb-seq to reveal the therapeutic response mechanism of glioblastoma.

The use of multi-omics techniques, including epigenomics, proteomics, metabolomics, and spatial genomics, at the single-cell level is expected to further enhance our understanding of the evolutionary landscape of tumor cells and facilitate the comprehensive identification of early tumor intervention or combination therapy targets [124-126]. For example, Savage *et al.* [127] successfully identified and validated potential targets for various cancer treatments by integrating multiple omics datasets. Researchers [128] have revealed distinct expression profiles of apoptosis-related genes in epidermal growth factor receptor (EGFR)- mutant lung cancer by integrating single-cell RNA sequencing (scRNA-seq) and spatial transcriptome analysis. With the development of single-cell technology, spatial multi-omics techniques have revealed cell interactions, microenvironments, and even systemic tumor-host interactions within the tumor ecosystem at the spatial level [129-131]. This not only enhances traditional therapies, such as surgery, radiotherapy, and chemotherapy, but also fosters the development of emerging targeted therapies for immunotherapy.

Tertiary lymphoid structure (TLS) is a special microscopic anatomical structure found in inflammatory tissues, similar to secondary lymphoid organs, with complex composition and structured tissues, and is particularly suitable for spatial research. It is associated with patient survival and can serve as a prognostic and predictive biomarker for immune therapy responses [132]. Research has shown that TLS has the potential to surpass the biomarkers currently used in clinical settings for immune checkpoint inhibitor (ICIs) responses. To gain a deeper understanding of the role of TLS in anti-tumor immunity, researchers have employed single-cell spatial transcriptomics to characterize the components of TLS and its precise function in the context of the TME [133, 134].

## 4 CHALLENGES AND PROSPECTS

The implementation of scRNA-seq technology requires complex experimental protocols and high-precision data analysis tools. Optimizing these protocols and ensuring data quality remain significant challenges [135-139]. For example, current efforts focus on reducing noise and signal loss during the experimental process, increasing sequencing throughput, and lowering costs. Future research requires the development of more precise, low-cost, and high-throughput single-cell sequencing technologies, as well as more efficient data analysis methods. Develop new single-cell separation technologies to improve the efficiency and purity of single-cell separation, and enhance sequencing depth and coverage. Improved quality control, data normalization algorithms, and optimized data integration solutions effectively integrate and utilize data from different platforms and laboratories, improving the utilization rate of existing data. Develop more accurate and reliable algorithms to process and interpret complex datasets, promoting their wider applications.

Combining proteomics, genomics, omics, and other multi-omics data can provide a more comprehensive understanding of the TME and drug action mechanisms, improving the accuracy of drug discovery. The integration of multiple omics and spatial transcriptomics will bring breakthroughs to tumor research.

Machine learning and deep learning are increasingly recognized for their capabilities in data analysis and processing, and play an increasingly important role in cancer research and pharmaceutical innovation. It can integrate large tumor datasets, decode the complex biological systems behind tumor development, deepen the understanding of drug-target interactions, optimize therapeutic strategies, and accelerate the process of identifying new drug candidates. Liangzhu Lab [140] and other teams have collaborated to launch an innovateive deep learning framework: "Shennong", which provides a new way to conduct computer-aided screening of antitumor drugs at the single-cell level. The Shennong framework takes a single-cell counting matrix and preprocessed cellular perturbation data as input. Using a deep learning model based on a variational self-encoder, the response strength (potential score) and change trend (potential direction) of each cell to a specific drug treatment are calculated. The final output is a matrix of scores for the effect of drug treatments on each cell. The variational autoencoder model consists of an encoder and a decoder, each containing four hidden layers. This method can not only predict the treatment and side effects of drugs and guide drug screening through output matrices, but also identify candidate drugs for specific cell types. By analyzing the weights of drug treatment-related genes in the model decoder, the relative importance of the gene in the corresponding drug treatment is explored, thus identifying potential targets of action for specific drugs. Through this model, researchers have identified FDA-approved drugs with new indications, candidate drugs undergoing clinical trials, and experimental compounds with anti-tumor activity. For example, the FDA-approved drug exemestane, used to treat breast cancer, has shown a significant anti-proliferation effect on lung cancer cells, and this discovery was confirmed in a Phase I clinical trial. The candidate drug “volasertib” has demonstrated safety and anti-tumor activity in multiple clinical trials and has the potential to alleviate the condition of non-small cell lung cancer patients. Cell line experiments have confirmed that the experimental compound “Parbendazole” exhibits anti-liver cancer activity. Therefore, there is a need to enhance the combination of artificial intelligence and single-cell transcriptome sequencing technologies in the future to improve the efficiency of drug discovery.

Although single-cell transcriptome sequencing technology has made significant progress in scientific research, it still faces substantial challenges for its widespread clinical application and true clinical translation, including technical costs, standardized procedures, clinical validation, and ethical and legal issues [141]. For example, in the application of clinical scRNA seq, data privacy issues are an extremely important and complex field. The research team must strike a balance between conducting scientific research and protecting patient privacy, ensuring compliance with relevant regulations, such as the General Data Protection Regulation (GDPR), while implementing effective de-identification strategies to maintain the security and privacy of patient data. Therefore, although a large amount of literature reveals the discovery of new potential drug targets, few ultimately enter the clinical development stage, and scientific research has not yet realized its full practical value. Promoting the transformation of tumor drug discovery from theoretical research to clinical development and final application, and advancing the application of single-cell transcriptome sequencing technology in clinical diagnosis and treatment, will significantly improve the treatment effectiveness for tumor patients.

## CONCLUSION

 Traditionally, researchers have obtained gene expression data from cell populations by averaging across the population. However, this approach masks the heterogeneity among different cells. The emergence of scRNA-seq technology has changed this situation. ScRNA-seq technology enables the acquisition of gene expression data at the cellular level. In addition, with the emergence of single-cell multi-omics and spatial transcriptomics, we can obtain more dimensional information about tumors. In this review, scRNA-seq technology has great potential for understanding diseases, identifying targets, high-throughput drug screening, and elucidating tumor drug resistance by revealing gene expression profiles and cellular heterogeneity in individual cells. Besides, this paper also highlights the technical and clinical challenges faced by scRNA-seq technology, offering new perspectives for the future development and application of this technology. It provides new ideas for tumor drug discovery and promotes the realization of precision medicine. This thesis reviews the relevant literature from the last few years; however, due to time and data limitations, this study has some shortcomings. In the future, it will continue to improve the research method to conduct in-depth research.

## AUTHORS’ CONTRIBUTIONS

The authors confirm their contribution to the paper as follows: study conception and design: Yueying Yang, Lingyu Meng, Teng Zhang and Jianjun Tan; data collection: Yueying Yang; analysis and interpretation of results: Yueying Yang; draft manuscript: Yueying Yangr. All authors reviewed the results and approved the final version of the manuscript.

## Figures and Tables

**Fig. (1):**
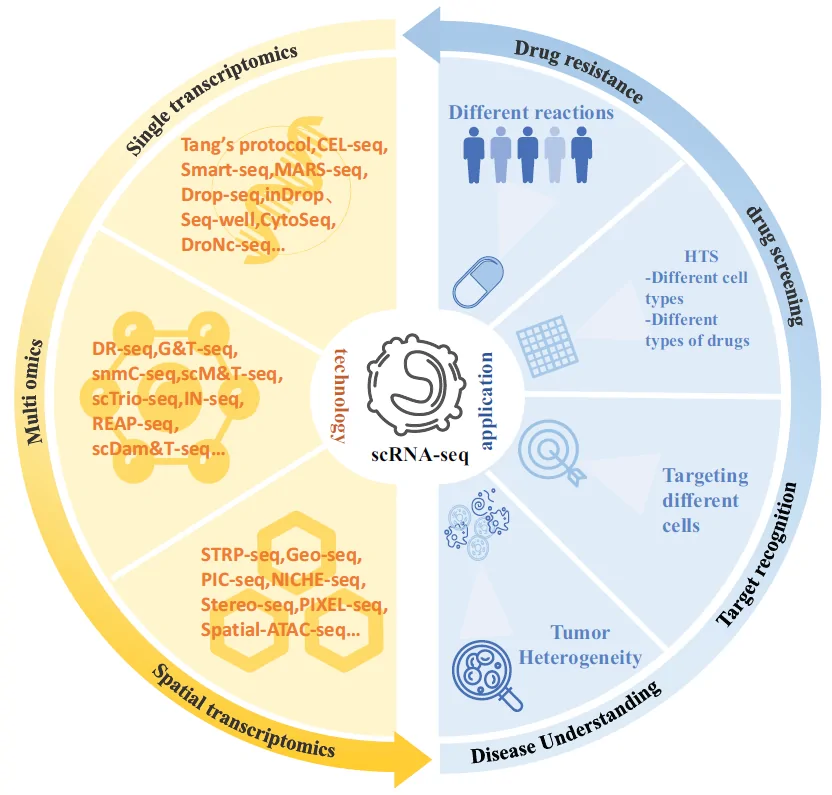
ScRNA-seq technology and its applications in tumor drug discovery. ScRNA-seq technology encompasses single-cell transcriptomics, multi-omics, and spatial transcriptomics. ScRNA-seq’s applications in tumor drug discovery include disease understanding, target recognition, drug screening, and analysis of drug resistance.

**Fig. (2):**
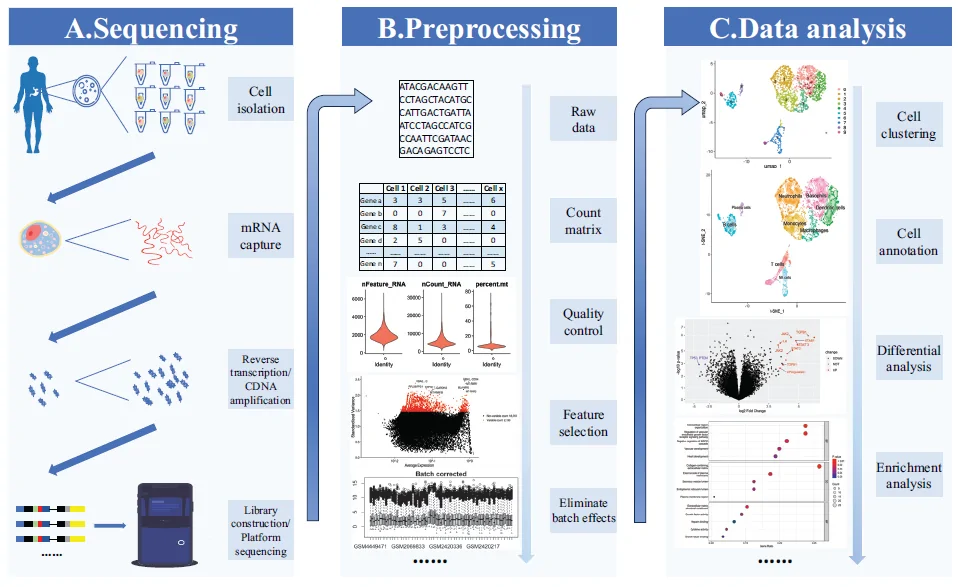
Single-Cell Sequencing Analysis. (**A**) Sequencing process involved single-cell isolation, mRNA capture, reverse transcription, cDNA amplification, library construction, and sorting. (**B**) Data preprocessing includes alignment, quality control, feature selection, and the elimination of batch effects. (**C**) Downstream data analysis involves cell clustering, annotation, difference analysis, and enrichment analysis.

## LIST OF ABBREVIATIONS

scRNA-seq: Single-cell RNA Sequencing
TME: Tumor Microenvironment
FASTQ: Fast Quality: A Text Format that Stores Biological Sequences (Usually Nucleic Acid Sequences) and Corresponding Quality Evaluations.
BCL: Base Call File
LCM: Laser Capture Microdissection
FACS: Fluorescence-activated Cell Sorting
UMIs: Unique Molecular Identifiers
GEO: Gene Expression Omnibus
HCA: Human Cell Atlas
CSCs: Cancer Stem Cells
CTCs: Circulating Tumor Cells
CAFs: Cancer-associated Fibroblasts
TNBC: Triple-negative Breast Cancer
EMT: Epithelial-mesenchymal Transition
CRISPR: Clustered Regularly Interspaced Short Palindromic Repeats
EGFR: Epidermal Growth Factor Receptor
TLS: Tertiary Lymphoid Structure
ICIs: Immune Checkpoint Inhibitors
GDPR: General Data Protection Regulation
FDA: The US Food and Drug Administration

## References

[1] Bray F., Laversanne M., Sung H., Ferlay J., Siegel R.L., Soerjomataram I., Jemal A. (2024). Global cancer statistics 2022: GLOBOCAN estimates of incidence and mortality worldwide for 36 cancers in 185 countries.. CA Cancer J. Clin..

[2] DiMasi J.A., Grabowski H.G., Hansen R.W. (2016). Innovation in the pharmaceutical industry: New estimates of R&D costs.. J. Health. Econ..

[3] Paul S.M., Mytelka D.S., Dunwiddie C.T., Persinger C.C., Munos B.H., Lindborg S.R., Schacht A.L. (2010). How to improve R&D productivity: The pharmaceutical industry’s grand challenge.. Nat. Rev. Drug Discov.

[4] Wang F., Ruan D.Y., Xu R.H. (2024). Challenges and opportunities in oncology drug development and clinical research in China.. Cell..

[5] Liu Z., Chen J., Ren Y., Liu S., Ba Y., Zuo A., Luo P., Cheng Q., Xu H., Han X. (2024). Multi-stage mechanisms of tumor metastasis and therapeutic strategies.. Signal. Transduct Target Ther..

[6] Zhang S., Xiao X., Yi Y., Wang X., Zhu L., Shen Y., Lin D., Wu C. (2024). Tumor initiation and early tumorigenesis: Molecular mechanisms and interventional targets.. Signal. Transduct Target Ther..

[7] El-Sayes N., Vito A., Mossman K. (2021). Tumor heterogeneity: A great barrier in the age of cancer immunotherapy.. Cancers.

[8] Imodoye S.O., Adedokun K.A., Bello I.O. (2024). From complexity to clarity: Unravelling tumor heterogeneity through the lens of tumor microenvironment for innovative cancer therapy.. Histochem. Cell. Biol..

[9] Duhan L., Kumari D., Naime M., Parmar V.S., Chhillar A.K., Dangi M., Pasrija R. (2024). Single-cell transcriptomics: Background, technologies, applications, and challenges.. Mol. Biol. Rep.

[10] Huang D., Ma N., Li X., Gou Y., Duan Y., Liu B., Xia J., Zhao X., Wang X., Li Q., Rao J., Zhang X. (2023). Advances in single-cell RNA sequencing and its applications in cancer research.. J. Hematol. Oncol..

[11] Liu B., Hu S., Wang X. (2024). Applications of single-cell technologies in drug discovery for tumor treatment.. iScience.

[12] Van de Sande B., Lee J.S., Mutasa-Gottgens E., Naughton B., Bacon W., Manning J., Wang Y., Pollard J., Mendez M., Hill J., Kumar N., Cao X., Chen X., Khaladkar M., Wen J., Leach A., Ferran E. (2023). Applications of single-cell RNA sequencing in drug discovery and development.. Nat. Rev. Drug Discov.

[13] Potter S.S. (2018). Single-cell RNA sequencing for the study of development, physiology and disease.. Nat. Rev. Nephrol.

[14] Gonzalez-Pena V., Gawad C. (2019). Sequencing the genomes of single cells.. Methods Mol. Biol..

[15] Lo P-K., Zhou Q. (2018). Emerging techniques in single-cell epigenomics and their applications to cancer research.. J. Clin. Genom.

[16] Stark R., Grzelak M., Hadfield J. (2019). RNA sequencing: The teenage years.. Nat. Rev. Genet..

[17] Wang Z., Gerstein M., Snyder M. (2009). RNA-Seq: A revolutionary tool for transcriptomics.. Nat. Rev. Genet..

[18] Chang X., Zheng Y., Xu K. (2024). Single-cell RNA sequencing: Technological progress and biomedical application in cancer research.. Mol. Biotechnol..

[19] Hwang B., Lee J.H., Bang D. (2018). Single-cell RNA sequencing technologies and bioinformatics pipelines.. Exp. Mol. Med..

[20] Luecken M.D., Theis F.J. (2019). Current best practices in single‐cell RNA‐seq analysis: A tutorial.. Mol. Syst. Biol..

[21] Nguyen Q.H., Pervolarakis N., Blake K., Ma D., Davis R.T., James N., Phung A.T., Willey E., Kumar R., Jabart E., Driver I., Rock J., Goga A., Khan S.A., Lawson D.A., Werb Z., Kessenbrock K. (2018). Profiling human breast epithelial cells using single cell RNA sequencing identifies cell diversity.. Nat. Commun.

[22] Tang F., Barbacioru C., Wang Y., Nordman E., Lee C., Xu N., Wang X., Bodeau J., Tuch B.B., Siddiqui A., Lao K., Surani M.A. (2009). mRNA-Seq whole-transcriptome analysis of a single cell.. Nat. Methods.

[23] Sun F.Y., Li H.Y., Sun D.Q., Fu S.L., Gu L., Shao X., Wang Q.Q., Dong X., Duan B., Xing F.Y., Wu J., Xiao M.M., Zhao F.Q., Han J.D.J., Liu Q., Fan X.H., Li C., Wang C.F., Shi T.L. (2024). Single-cell omics: Experimental workflow, data analyses and applications.. Sci. China Life. Sci..

[24] Gross A., Schoendube J., Zimmermann S., Steeb M., Zengerle R., Koltay P. (2015). Technologies for single-cell isolation.. Int. J. Mol. Sci..

[25] Zhang X., Wei X., Wei Y., Chen M., Wang J. (2020). The up-to-date strategies for the isolation and manipulation of single cells.. Talanta.

[26] Gautam V., Chatterjee S., Sarkar A.K. (2021). Single cell type specific RNA isolation and gene expression analysis in rice using laser capture microdissection (LCM)-based method.. Methods Mol. Biol..

[27] Bowers R.M., Lee J., Woyke T. (2018). Sequencing of genomes from environmental single cells.. Methods Mol. Biol..

[28] Gassei K., Ehmcke J., Schlatt S. (2009). Efficient enrichment of undifferentiated GFR alpha 1+ spermatogonia from immature rat testis by magnetic activated cell sorting.. Cell. Tissue Res..

[29] García Alonso D., Yu M., Qu H., Ma L., Shen F. (2019). Advances in microfluidics‐based technologies for single cell culture.. Adv. Biosyst.

[30] Lan Y., Zhou Y., Wu M., Jia C., Zhao J. (2023). Microfluidic based single cell or droplet manipulation: Methods and applications.. Talanta.

[31] Salmen F., De Jonghe J., Kaminski T.S., Alemany A., Parada G.E., Verity-Legg J., Yanagida A., Kohler T.N., Battich N., van den Brekel F., Ellermann A.L., Arias A.M., Nichols J., Hemberg M., Hollfelder F., van Oudenaarden A. (2022). High-throughput total RNA sequencing in single cells using VASA-seq.. Nat. Biotechnol..

[32] Ambardar S., Gupta R., Trakroo D., Lal R., Vakhlu J. (2016). High throughput sequencing: An overview of sequencing chemistry.. Indian J. Microbiol..

[33] Hagemann-Jensen M., Ziegenhain C., Sandberg R. (2022). Scalable single-cell RNA sequencing from full transcripts with Smart-seq3xpress.. Nat. Biotechnol..

[34] Ramsköld D., Luo S., Wang Y.C., Li R., Deng Q., Faridani O.R., Daniels G.A., Khrebtukova I., Loring J.F., Laurent L.C., Schroth G.P., Sandberg R. (2012). Full-length mRNA-Seq from single-cell levels of RNA and individual circulating tumor cells.. Nat. Biotechnol..

[35] Fan X., Tang D., Liao Y., Li P., Zhang Y., Wang M., Liang F., Wang X., Gao Y., Wen L., Wang D., Wang Y., Tang F. (2020). Single-cell RNA-seq analysis of mouse preimplantation embryos by third-generation sequencing.. PLoS Biol..

[36] Liao Y., Liu Z., Zhang Y., Lu P., Wen L., Tang F. (2023). High-throughput and high-sensitivity full-length single-cell RNA-seq analysis on third-generation sequencing platform.. Cell. Discov.

[37] Jaitin D.A., Kenigsberg E., Keren-Shaul H., Elefant N., Paul F., Zaretsky I., Mildner A., Cohen N., Jung S., Tanay A., Amit I. (2014). Massively parallel single-cell RNA-seq for marker-free decomposition of tissues into cell types.. Science.

[38] Hashimshony T., Wagner F., Sher N., Yanai I. (2012). CEL-Seq: single-cell RNA-Seq by multiplexed linear amplification.. Cell. Rep.

[39] Hashimshony T., Senderovich N., Avital G., Klochendler A., de Leeuw Y., Anavy L., Gennert D., Li S., Livak K.J., Rozenblatt-Rosen O., Dor Y., Regev A., Yanai I. (2016). CEL-Seq2: Sensitive highly-multiplexed single-cell RNA-Seq.. Genome Biol..

[40] Islam S., Kjällquist U., Moliner A., Zajac P., Fan J.B., Lönnerberg P., Linnarsson S. (2011). Characterization of the single-cell transcriptional landscape by highly multiplex RNA-seq.. Genome Res..

[41] Islam S., Zeisel A., Joost S., La Manno G., Zajac P., Kasper M., Lönnerberg P., Linnarsson S. (2014). Quantitative single-cell RNA-seq with unique molecular identifiers.. Nat. Methods.

[42] Zilionis R., Nainys J., Veres A., Savova V., Zemmour D., Klein A.M., Mazutis L. (2017). Single-cell barcoding and sequencing using droplet microfluidics.. Nat. Protoc.

[43] Gierahn T.M., Wadsworth M.H., Hughes T.K., Bryson B.D., Butler A., Satija R., Fortune S., Love J.C., Shalek A.K. (2017). Seq-Well: Portable, low-cost RNA sequencing of single cells at high throughput.. Nat. Methods.

[44] Macosko E.Z., Basu A., Satija R., Nemesh J., Shekhar K., Goldman M., Tirosh I., Bialas A.R., Kamitaki N., Martersteck E.M., Trombetta J.J., Weitz D.A., Sanes J.R., Shalek A.K., Regev A., McCarroll S.A. (2015). Highly parallel genome-wide expression profiling of individual cells using nanoliter droplets.. Cell..

[45] Klein A.M., Mazutis L., Akartuna I., Tallapragada N., Veres A., Li V., Peshkin L., Weitz D.A., Kirschner M.W. (2015). Droplet barcoding for single-cell transcriptomics applied to embryonic stem cells.. Cell..

[46] Baysoy A., Bai Z., Satija R., Fan R. (2023). The technological landscape and applications of single-cell multi-omics.. Nat. Rev. Mol. Cell. Biol..

[47] Wu X., Yang X., Dai Y., Zhao Z., Zhu J., Guo H., Yang R. (2024). Single-cell sequencing to multi-omics: Technologies and applications.. Biomark Res..

[48] Macaulay I.C., Haerty W., Kumar P., Li Y.I., Hu T.X., Teng M.J., Goolam M., Saurat N., Coupland P., Shirley L.M., Smith M., Van der Aa N., Banerjee R., Ellis P.D., Quail M.A., Swerdlow H.P., Zernicka-Goetz M., Livesey F.J., Ponting C.P., Voet T. (2015). G&T-seq: Parallel sequencing of single-cell genomes and transcriptomes.. Nat. Methods.

[49] Dey S.S., Kester L., Spanjaard B., Bienko M., van Oudenaarden A. (2015). Integrated genome and transcriptome sequencing of the same cell.. Nat. Biotechnol..

[50] Hou Y., Guo H., Cao C., Li X., Hu B., Zhu P., Wu X., Wen L., Tang F., Huang Y., Peng J. (2016). Single-cell triple omics sequencing reveals genetic, epigenetic, and transcriptomic heterogeneity in hepatocellular carcinomas.. Cell. Res..

[51] Angermueller C., Clark S.J., Lee H.J., Macaulay I.C., Teng M.J., Hu T.X., Krueger F., Smallwood S.A., Ponting C.P., Voet T., Kelsey G., Stegle O., Reik W. (2016). Parallel single-cell sequencing links transcriptional and epigenetic heterogeneity.. Nat. Methods.

[52] Stoeckius M., Hafemeister C., Stephenson W., Houck-Loomis B., Chattopadhyay P.K., Swerdlow H., Satija R., Smibert P. (2017). Simultaneous epitope and transcriptome measurement in single cells.. Nat. Methods.

[53] Peterson V.M., Zhang K.X., Kumar N., Wong J., Li L., Wilson D.C., Moore R., McClanahan T.K., Sadekova S., Klappenbach J.A. (2017). Multiplexed quantification of proteins and transcripts in single cells.. Nat. Biotechnol..

[54] Rooijers K., Markodimitraki C.M., Rang F.J., de Vries S.S., Chialastri A., de Luca K.L., Mooijman D., Dey S.S., Kind J. (2019). Simultaneous quantification of protein–DNA contacts and transcriptomes in single cells.. Nat. Biotechnol..

[55] Chen S., Lake B.B., Zhang K. (2019). High-throughput sequencing of the transcriptome and chromatin accessibility in the same cell.. Nat. Biotechnol..

[56] Liu L., Liu C., Quintero A., Wu L., Yuan Y., Wang M., Cheng M., Leng L., Xu L., Dong G., Li R., Liu Y., Wei X., Xu J., Chen X., Lu H., Chen D., Wang Q., Zhou Q., Lin X., Li G., Liu S., Wang Q., Wang H., Fink J.L., Gao Z., Liu X., Hou Y., Zhu S., Yang H., Ye Y., Lin G., Chen F., Herrmann C., Eils R., Shang Z., Xu X. (2019). Deconvolution of single-cell multi-omics layers reveals regulatory heterogeneity.. Nat. Commun.

[57] Wang Y., Yuan P., Yan Z., Yang M., Huo Y., Nie Y., Zhu X., Qiao J., Yan L. (2021). Single-cell multiomics sequencing reveals the functional regulatory landscape of early embryos.. Nat. Commun.

[58] Luo C., Liu H., Xie F., Armand E.J., Siletti K., Bakken T.E., Fang R., Doyle W.I., Stuart T., Hodge R.D., Hu L., Wang B.A., Zhang Z., Preissl S., Lee D.S., Zhou J., Niu S.Y., Castanon R., Bartlett A., Rivkin A., Wang X., Lucero J., Nery J.R., Davis D.A., Mash D.C., Satija R., Dixon J.R., Linnarsson S., Lein E., Behrens M.M., Ren B., Mukamel E.A., Ecker J.R. (2022). Single nucleus multi-omics identifies human cortical cell regulatory genome diversity.. Cell. Genomics.

[59] Mimitou E.P., Cheng A., Montalbano A., Hao S., Stoeckius M., Legut M., Roush T., Herrera A., Papalexi E., Ouyang Z., Satija R., Sanjana N.E., Koralov S.B., Smibert P. (2019). Multiplexed detection of proteins, transcriptomes, clonotypes and CRISPR perturbations in single cells.. Nat. Methods.

[60] Clark S.J., Argelaguet R., Kapourani C.A., Stubbs T.M., Lee H.J., Alda-Catalinas C., Krueger F., Sanguinetti G., Kelsey G., Marioni J.C., Stegle O., Reik W. (2018). scNMT-seq enables joint profiling of chromatin accessibility DNA methylation and transcription in single cells.. Nat. Commun.

[61] Mimitou E.P., Lareau C.A., Chen K.Y., Zorzetto-Fernandes A.L., Hao Y., Takeshima Y., Luo W., Huang T.S., Yeung B.Z., Papalexi E., Thakore P.I., Kibayashi T., Wing J.B., Hata M., Satija R., Nazor K.L., Sakaguchi S., Ludwig L.S., Sankaran V.G., Regev A., Smibert P. (2021). Scalable, multimodal profiling of chromatin accessibility, gene expression and protein levels in single cells.. Nat. Biotechnol..

[62] Li X., Wang C.Y. (2021). From bulk, single-cell to spatial RNA sequencing.. Int. J. Oral Sci..

[63] Kruse F., Junker J.P., van Oudenaarden A., Bakkers J. (2016). Tomo-seq: A method to obtain genome-wide expression data with spatial resolution.. Methods Cell. Biol..

[64] Lovatt D., Ruble B.K., Lee J., Dueck H., Kim T.K., Fisher S., Francis C., Spaethling J.M., Wolf J.A., Grady M.S., Ulyanova A.V., Yeldell S.B., Griepenburg J.C., Buckley P.T., Kim J., Sul J.Y., Dmochowski I.J., Eberwine J. (2014). Transcriptome in vivo analysis (TIVA) of spatially defined single cells in live tissue.. Nat. Methods.

[65] Lee J.H., Daugharthy E.R., Scheiman J., Kalhor R., Ferrante T.C., Terry R., Turczyk B.M., Yang J.L., Lee H.S., Aach J., Zhang K., Church G.M. (2015). Fluorescent in situ sequencing (FISSEQ) of RNA for gene expression profiling in intact cells and tissues.. Nat. Protoc.

[66] Medaglia C., Giladi A., Stoler-Barak L., De Giovanni M., Salame T.M., Biram A., David E., Li H., Iannacone M., Shulman Z., Amit I. (2017). Spatial reconstruction of immune niches by combining photoactivatable reporters and scRNA-seq.. Science.

[67] Chen J., Suo S., Tam P.P.L., Han J.D.J., Peng G., Jing N. (2017). Spatial transcriptomic analysis of cryosectioned tissue samples with Geo-seq.. Nat. Protoc.

[68] Cai L. (2020). Transcriptome-scale super-resolved imaging in tissues by RNA SeqFISH.. Eur J. Hum. Genet..

[69] Giladi A., Cohen M., Medaglia C., Baran Y., Li B., Zada M., Bost P., Blecher-Gonen R., Salame T.M., Mayer J.U., David E., Ronchese F., Tanay A., Amit I. (2020). Dissecting cellular crosstalk by sequencing physically interacting cells.. Nat. Biotechnol..

[70] Fu X., Sun L., Dong R., Chen J.Y., Silakit R., Condon L.F., Lin Y., Lin S., Palmiter R.D., Gu L. (2022). Polony gels enable amplifiable DNA stamping and spatial transcriptomics of chronic pain.. Cell..

[71] Ståhl P.L., Salmén F., Vickovic S., Lundmark A., Navarro J.F., Magnusson J., Giacomello S., Asp M., Westholm J.O., Huss M., Mollbrink A., Linnarsson S., Codeluppi S., Borg Å., Pontén F., Costea P.I., Sahlén P., Mulder J., Bergmann O., Lundeberg J., Frisén J. (2016). Visualization and analysis of gene expression in tissue sections by spatial transcriptomics.. Science.

[72] Lee J.H., Daugharthy E.R., Scheiman J., Kalhor R., Yang J.L., Ferrante T.C., Terry R., Jeanty S.S.F., Li C., Amamoto R., Peters D.T., Turczyk B.M., Marblestone A.H., Inverso S.A., Bernard A., Mali P., Rios X., Aach J., Church G.M. (2014). Highly multiplexed subcellular RNA sequencing in situ.. Science.

[73] Schede H.H., Schneider C.G., Stergiadou J., Borm L.E., Ranjak A., Yamawaki T.M., David F.P.A., Lönnerberg P., Tosches M.A., Codeluppi S., La Manno G. (2021). Spatial tissue profiling by imaging-free molecular tomography.. Nat. Biotechnol..

[74] Chen A., Liao S., Cheng M., Ma K., Wu L., Lai Y., Qiu X., Yang J., Xu J., Hao S., Wang X., Lu H., Chen X., Liu X., Huang X., Li Z., Hong Y., Jiang Y., Peng J., Liu S., Shen M., Liu C., Li Q., Yuan Y., Wei X., Zheng H., Feng W., Wang Z., Liu Y., Wang Z., Yang Y., Xiang H., Han L., Qin B., Guo P., Lai G., Muñoz-Cánoves P., Maxwell P.H., Thiery J.P., Wu Q.F., Zhao F., Chen B., Li M., Dai X., Wang S., Kuang H., Hui J., Wang L., Fei J.F., Wang O., Wei X., Lu H., Wang B., Liu S., Gu Y., Ni M., Zhang W., Mu F., Yin Y., Yang H., Lisby M., Cornall R.J., Mulder J., Uhlén M., Esteban M.A., Li Y., Liu L., Xu X., Wang J. (2022). Spatiotemporal transcriptomic atlas of mouse organogenesis using DNA nanoball-patterned arrays.. Cell..

[75] Hao Y., Stuart T., Kowalski M.H., Choudhary S., Hoffman P., Hartman A., Srivastava A., Molla G., Madad S., Fernandez-Granda C., Satija R. (2024). Dictionary learning for integrative, multimodal and scalable single-cell analysis.. Nat. Biotechnol..

[76] Wolf F.A., Angerer P., Theis F.J. (2018). SCANPY: Large-scale single-cell gene expression data analysis.. Genome Biol..

[77] Edgar R., Domrachev M., Lash A.E. (2002). Gene Expression Omnibus: NCBI gene expression and hybridization array data repository.. Nucleic Acids Res..

[78] Regev A., Teichmann S.A., Lander E.S., Amit I., Benoist C., Birney E., Bodenmiller B., Campbell P., Carninci P., Clatworthy M., Clevers H., Deplancke B., Dunham I., Eberwine J., Eils R., Enard W., Farmer A., Fugger L., Göttgens B., Hacohen N., Haniffa M., Hemberg M., Kim S., Klenerman P., Kriegstein A., Lein E., Linnarsson S., Lundberg E., Lundeberg J., Majumder P., Marioni J.C., Merad M., Mhlanga M., Nawijn M., Netea M., Nolan G., Pe’er D., Phillipakis A., Ponting C.P., Quake S., Reik W., Rozenblatt-Rosen O., Sanes J., Satija R., Schumacher T.N., Shalek A., Shapiro E., Sharma P., Shin J.W., Stegle O., Stratton M., Stubbington M.J.T., Theis F.J., Uhlen M., van Oudenaarden A., Wagner A., Watt F., Weissman J., Wold B., Xavier R., Yosef N. (2017). The Human Cell Atlas.. eLife.

[79] Cao Y., Zhu J., Jia P., Zhao Z. (2017). scRNASeqDB: A database for RNA-Seq based gene expression profiles in human single cells.. Genes.

[80] Li Y., Ma L., Wu D., Chen G. (2021). Advances in bulk and single-cell multi-omics approaches for systems biology and precision medicine.. Brief Bioinform..

[81] Liu Q., Liang Y., Wang D., Li J. (2022). LFSC: A linear fast semi-supervised clustering algorithm that integrates reference-bulk and single-cell transcriptomes.. Front Genet..

[82] Jew B., Alvarez M., Rahmani E., Miao Z., Ko A., Garske K.M., Sul J.H., Pietiläinen K.H., Pajukanta P., Halperin E. (2020). Accurate estimation of cell composition in bulk expression through robust integration of single-cell information.. Nat. Commun.

[83] Awuah W.A., Roy S., Tan J.K., Adebusoye F.T., Qiang Z., Ferreira T., Ahluwalia A., Shet V., Yee A.L.W., Abdul-Rahman T., Papadakis M. (2024). Exploring the current landscape of single‐cell RNA sequencing applications in gastric cancer research.. J. Cell. Mol. Med..

[84] Nicholas C.A., Smith M.J. (2023). Application of single-cell RNA sequencing methods to develop B cell targeted treatments for autoimmunity.. Front Immunol..

[85] Cao Y.H., Ding J., Tang Q.H., Zhang J., Huang Z.Y., Tang X.M., Liu J.B., Ma Y.S., Fu D. (2022). Deciphering cell–cell interactions and communication in the tumor microenvironment and unraveling intratumoral genetic heterogeneity via single-cell genomic sequencing.. Bioengineered.

[86] Li P.H., Kong X.Y., He Y.Z., Liu Y., Peng X., Li Z.H., Xu H., Luo H., Park J. (2022). Recent developments in application of single-cell RNA sequencing in the tumour immune microenvironment and cancer therapy.. Mil Med. Res..

[87] Greten F.R., Grivennikov S., Newman A.M., Astle W.J. (2024). Genomic map of the cellular composition of the tumor microenvironment.. Nat. Immunol..

[88] Cheng S., Li Z., Gao R., Xing B., Gao Y., Yang Y., Qin S., Zhang L., Ouyang H., Du P., Jiang L., Zhang B., Yang Y., Wang X., Ren X., Bei J.X., Hu X., Bu Z., Ji J., Zhang Z. (2021). A pan-cancer single-cell transcriptional atlas of tumor infiltrating myeloid cells.. Cell..

[89] Tang F., Li J., Qi L., Liu D., Bo Y., Qin S., Miao Y., Yu K., Hou W., Li J., Peng J., Tian Z., Zhu L., Peng H., Wang D., Zhang Z. (2023). A pan-cancer single-cell panorama of human natural killer cells.. Cell..

[90] Zhang L., Yu X., Zheng L., Zhang Y., Li Y., Fang Q., Gao R., Kang B., Zhang Q., Huang J.Y., Konno H., Guo X., Ye Y., Gao S., Wang S., Hu X., Ren X., Shen Z., Ouyang W., Zhang Z. (2018). Lineage tracking reveals dynamic relationships of T cells in colorectal cancer.. Nature.

[91] Zhang Q., He Y., Luo N., Patel S.J., Han Y., Gao R., Modak M., Carotta S., Haslinger C., Kind D., Peet G.W., Zhong G., Lu S., Zhu W., Mao Y., Xiao M., Bergmann M., Hu X., Kerkar S.P., Vogt A.B., Pflanz S., Liu K., Peng J., Ren X., Zhang Z. (2019). Landscape and dynamics of single immune cells in hepatocellular carcinoma.. Cell.

[92] Zheng C., Zheng L., Yoo J.K., Guo H., Zhang Y., Guo X., Kang B., Hu R., Huang J.Y., Zhang Q., Liu Z., Dong M., Hu X., Ouyang W., Peng J., Zhang Z. (2017). Landscape of infiltrating T cells in liver cancer revealed by single-cell sequencing.. Cell..

[93] Zhao Q., Zhao R., Song C., Wang H., Rong J., Wang F., Yan L., Song Y., Xie Y. (2021). Increased IGFBP7 expression correlates with poor prognosis and immune infiltration in gastric cancer.. J. Cancer.

[94] Yang J., Wu B., Li G., Zhang C., Xie Y., Kong W., Zeng Z. (2024). Landscape of epithelial cell subpopulations in the human esophageal squamous cell carcinoma microenvironment.. Heliyon.

[95] Wilk A.J., Shalek A.K., Holmes S., Blish C.A. (2024). Comparative analysis of cell–cell communication at single-cell resolution.. Nat. Biotechnol..

[96] Li L., Liu J., Wang W., Fu Y., Deng Y., Li X., Liu Z., Pang Y., Xu Y., Yan M., Li Z. (2023). Cancer stem cells promote lymph nodes metastasis of breast cancer by reprogramming tumor microenvironment.. Transl. Oncol..

[97] Yao J., Liu Y., Yang J., Li M., Li S., Zhang B., Yang R., Zhang Y., Cui X., Feng C. (2022). Single-cell sequencing reveals that DBI is the key gene and potential therapeutic target in quiescent bladder cancer stem cells.. Front Genet..

[98] Orrapin S., Thongkumkoon P., Udomruk S., Moonmuang S., Sutthitthasakul S., Yongpitakwattana P., Pruksakorn D., Chaiyawat P. (2023). Deciphering the biology of circulating tumor cells through single-cell RNA sequencing: Implications for precision medicine in cancer.. Int. J. Mol. Sci..

[99] Gostomczyk K., Marsool M.D.M., Tayyab H., Pandey A., Borowczak J., Macome F., Chacon J., Dave T., Maniewski M., Szylberg Ł. (2023). Targeting circulating tumor cells to prevent metastases.. Hum. Cell..

[100] Chen C., Guo Q., Liu Y., Hou Q., Liao M., Guo Y., Zang Y., Wang F., Liu H., Luan X., Liang Y., Guan Z., Li Y., Liu H., Dong X., Zhang X., Liu J., Xu Q. (2023). Single‐cell and spatial transcriptomics reveal POSTN+ cancer‐associated fibroblasts correlated with immune suppression and tumour progression in non‐small cell lung cancer.. Clin. Transl. Med..

[101] Rui R., Zhou L., He S. (2023). Cancer immunotherapies: Advances and bottlenecks.. Front Immunol..

[102] Luo W., Zeng Z., Jin Y., Yang L., Fan T., Wang Z., Pan Y., Yang Y., Yao M., Li Y., Xiao X., Wang G., Wang C., Chang S., Che G., Zhang L., Li Y., Peng Y., Li W. (2023). Distinct immune microenvironment of lung adenocarcinoma in never-smokers from smokers.. Cell. Rep Med..

[103] Bock C., Datlinger P., Chardon F., Coelho M.A., Dong M.B., Lawson K.A., Lu T., Maroc L., Norman T.M., Song B., Stanley G., Chen S., Garnett M., Li W., Moffat J., Qi L.S., Shapiro R.S., Shendure J., Weissman J.S., Zhuang X. (2022). High-content CRISPR screening.. Nat. Rev. Methods Primers.

[104] Lotfollahi M., Klimovskaia Susmelj A., De Donno C., Hetzel L., Ji Y., Ibarra I.L., Srivatsan S.R., Naghipourfar M., Daza R.M., Martin B., Shendure J., McFaline-Figueroa J.L., Boyeau P., Wolf F.A., Yakubova N., Günnemann S., Trapnell C., Lopez-Paz D., Theis F.J. (2023). Predicting cellular responses to complex perturbations in high‐throughput screens.. Mol. Syst. Biol..

[105] Gezelius H., Enblad A.P., Lundmark A., Åberg M., Blom K., Rudfeldt J., Raine A., Harila A., Rendo V., Heinäniemi M., Andersson C., Nordlund J. (2024). Comparison of high-throughput single-cell RNA-seq methods for ex vivo drug screening.. NAR Genom Bioinform..

[106] Shin D., Lee W., Lee J.H., Bang D. (2019). Multiplexed single-cell RNA-seq via transient barcoding for simultaneous expression profiling of various drug perturbations.. Sci. Adv..

[107] Duan X., Zhang T., Feng L., de Silva N., Greenspun B., Wang X., Moyer J., Martin M.L., Chandwani R., Elemento O., Leach S.D., Evans T., Chen S., Pan F.C. (2024). A pancreatic cancer organoid platform identifies an inhibitor specific to mutant KRAS.. Cell. Stem Cell..

[108] McFarland J.M., Paolella B.R., Warren A., Geiger-Schuller K., Shibue T., Rothberg M., Kuksenko O., Colgan W.N., Jones A., Chambers E., Dionne D., Bender S., Wolpin B.M., Ghandi M., Tirosh I., Rozenblatt-Rosen O., Roth J.A., Golub T.R., Regev A., Aguirre A.J., Vazquez F., Tsherniak A. (2020). Multiplexed single-cell transcriptional response profiling to define cancer vulnerabilities and therapeutic mechanism of action.. Nat. Commun.

[109] Li C., Shao X., Zhang S., Wang Y., Jin K., Yang P., Lu X., Fan X., Wang Y. (2024). scRank infers drug-responsive cell types from untreated scRNA-seq data using a target-perturbed gene regulatory network.. Cell. Rep Med..

[110] Chen D., Liu P., Lu X., Li J., Qi D., Zang L., Lin J., Liu Y., Zhai S., Fu D., Weng Y., Li H., Shen B. (2024). Pan-cancer analysis implicates novel insights of lactate metabolism into immunotherapy response prediction and survival prognostication.. J. Exp. Clin. Cancer Res..

[111] Foutadakis S., Kordias D., Vatsellas G., Magklara A. (2024). Identification of new chemoresistance-associated genes in triple-negative breast cancer by single-cell transcriptomic analysis.. Int. J. Mol. Sci..

[112] Fateeva A., Chen S. (2023). Editorial: The role of immunotherapy in melanomas.. Front Oncol..

[113] Premi S., Qin Y., Ahmad N. (2022). Editorial: Mechanisms of resistance to the targeted therapy and immunotherapy in cutaneous melanoma.. Front Oncol..

[114] Spiliopoulou P., Vornicova O., Genta S., Spreafico A. (2023). Shaping the future of immunotherapy targets and biomarkers in melanoma and non-melanoma cutaneous cancers.. Int. J. Mol. Sci..

[115] Ding Y., Zhao Z., Cai H., Zhou Y., Chen H., Bai Y., Liu Z., Liu S., Zhou W. (2023). Single-cell sequencing analysis related to sphingolipid metabolism guides immunotherapy and prognosis of skin cutaneous melanoma.. Front Immunol..

[116] Tang S., Zhang Y., Huang S., Zhu T., Huang X. (2024). Single cell RNA-sequencing in uveal melanoma: Advances in heterogeneity, tumor microenvironment and immunotherapy.. Front Immunol..

[117] He B., Xiao Y., Liang H., Huang Q., Du Y., Li Y., Garmire D., Sun D., Garmire L.X. (2023). ASGARD is a single-cell guided pipeline to aid repurposing of drugs.. Nat. Commun.

[118] Hsieh C.Y., Wen J.H., Lin S.M., Tseng T.Y., Huang J.H., Huang H.C., Juan H.F. (2023). scDrug: From single-cell RNA-seq to drug response prediction.. Comput. Struct. Biotechnol. J..

[119] Sun Y.Y., Hsieh C.Y., Wen J.H., Tseng T.Y., Huang J.H., Oyang Y.J., Huang H.C., Juan H.F. (2024). scDrug+: Predicting drug-responses using single-cell transcriptomics and molecular structure.. Biomed. Pharmacother.

[120] Lee H.M., Wright W.C., Pan M., Low J., Currier D., Fang J., Singh S., Nance S., Delahunty I., Kim Y., Chapple R.H., Zhang Y., Liu X., Steele J.A., Qi J., Pruett-Miller S.M., Easton J., Chen T., Yang J., Durbin A.D., Geeleher P. (2023). A CRISPR-drug perturbational map for identifying compounds to combine with commonly used chemotherapeutics.. Nat. Commun.

[121] Zhong C., Jiang W.J., Yao Y., Li Z., Li Y., Wang S., Wang X., Zhu W., Wu S., Wang J., Fan S., Ma S., Liu Y., Zhang H., Zhao W., Zhao L., Feng Y., Li Z., Guo R., Yu L., Pei F., Hu J., Feng X., Yang Z., Yang Z., Yang X., Hou Y., Zhang D., Xu D., Sheng R., Li Y., Liu L., Wu H.J., Huang J., Fei T. (2024). CRISPR screens reveal convergent targeting strategies against evolutionarily distinct chemoresistance in cancer.. Nat. Commun.

[122] Jin X., Simmons S.K., Guo A., Shetty A.S., Ko M., Nguyen L., Jokhi V., Robinson E., Oyler P., Curry N., Deangeli G., Lodato S., Levin J.Z., Regev A., Zhang F., Arlotta P. (2020). In vivo Perturb-Seq reveals neuronal and glial abnormalities associated with autism risk genes.. Science.

[123] Liu S.J., Zou C., Pak J., Morse A., Pang D., Casey-Clyde T., Borah A.A., Wu D., Seo K., O’Loughlin T., Lim D.A., Ozawa T., Berger M.S., Kamber R.A., Weiss W.A., Raleigh D.R., Gilbert L.A. (2024). In vivo perturb-seq of cancer and microenvironment cells dissects oncologic drivers and radiotherapy responses in glioblastoma.. Genome Biol..

[124] Janesick A., Shelansky R., Gottscho A.D., Wagner F., Williams S.R., Rouault M., Beliakoff G., Morrison C.A., Oliveira M.F., Sicherman J.T., Kohlway A., Abousoud J., Drennon T.Y., Mohabbat S.H., Taylor S.E.B. (2023). High resolution mapping of the tumor microenvironment using integrated single-cell, spatial and in situ analysis.. Nat. Commun.

[125] Liang A., Kong Y., Chen Z., Qiu Y., Wu Y., Zhu X., Li Z. (2024). Advancements and applications of single-cell multi-omics techniques in cancer research: Unveiling heterogeneity and paving the way for precision therapeutics.. Biochem. Biophys. Rep.

[126] Wang Q., Zhi Y., Zi M., Mo Y., Wang Y., Liao Q., Zhang S., Gong Z., Wang F., Zeng Z., Guo C., Xiong W. (2023). Spatially resolved transcriptomics technology facilitates cancer research.. Adv. Sci..

[127] Savage S.R., Yi X., Lei J.T., Wen B., Zhao H., Liao Y., Jaehnig E.J., Somes L.K., Shafer P.W., Lee T.D., Fu Z., Dou Y., Shi Z., Gao D., Hoyos V., Gao Q., Zhang B. (2024). Pan-cancer proteogenomics expands the landscape of therapeutic targets.. Cell..

[128] Izumi M., Fujii M., Kobayashi I.S., Ho V., Kashima Y., Udagawa H., Costa D.B., Kobayashi S.S. (2024). Integrative single-cell RNA-seq and spatial transcriptomics analyses reveal diverse apoptosis-related gene expression profiles in EGFR-mutated lung cancer.. Cell. Death Dis..

[129] Liu Y., Yang M., Deng Y., Su G., Enninful A., Guo C.C., Tebaldi T., Zhang D., Kim D., Bai Z., Norris E., Pan A., Li J., Xiao Y., Halene S., Fan R. (2020). High-spatial-resolution multi-omics sequencing via deterministic barcoding in tissue.. Cell..

[130] Jiang F., Zhou X., Qian Y., Zhu M., Wang L., Li Z., Shen Q., Wang M., Qu F., Cui G., Chen K., Peng G. (2023). Simultaneous profiling of spatial gene expression and chromatin accessibility during mouse brain development.. Nat. Methods.

[131] Wu X., Xu W., Deng L., Li Y., Wang Z., Sun L., Gao A., Wang H., Yang X., Wu C., Zou Y., Yan K., Liu Z., Zhang L., Du G., Yang L., Lin D., Yue J., Wang P., Han Y., Fu Z., Dai J., Cao G. (2024). Spatial multi-omics at subcellular resolution via high-throughput in situ pairwise sequencing.. Nat. Biomed. Eng..

[132] Chen J., Larsson L., Swarbrick A., Lundeberg J. (2024). Spatial landscapes of cancers: Insights and opportunities.. Nat. Rev. Clin. Oncol..

[133] Li S., Wang Z., Huang H.D., Lee T.Y. (2024). Machine learning-based characterization and identification of tertiary lymphoid structures using spatial transcriptomics data.. Int. J. Mol. Sci..

[134] Xu W., Lu J., Tian X., Ye S., Wei S., Wang J., Anwaier A., Qu Y., Liu W., Chang K., Zhang H., Ye D. (2024). Unveiling the impact of tertiary lymphoid structures on immunotherapeutic responses of clear cell renal cell carcinoma.. MedComm.

[135] Jiang P. (2019). Quality control of single-cell RNA-seq.. Methods Mol. Biol..

[136] Qu H.Q., Kao C., Hakonarson H. (2024). Single-cell RNA sequencing technology landscape in 2023.. Stem Cells.

[137] Xiong J., Gong F., Ma L., Wan L. (2024). scVIC: Deep generative modeling of heterogeneity for scRNA-seq data.. Bioinform. Adv..

[138] Xu L., Li Z., Ren J., Liu S., Xu Y. (2024). Single-cell RNA sequencing data analysis utilizing multi-type graph neural networks.. Comput. Biol. Med..

[139] Yarlagadda S., Giorgio T.D. (2024). A guide to single‐cell RNA sequencing analysis using web‐based tools for non‐bioinformatician.. FEBS J..

[140] Zhang P., Wang X., Cen X., Zhang Q., Fu Y., Mei Y., Wang X., Wang R., Wang J., Ouyang H., Liang T., Xia H., Han X., Guo G. (2025). A deep learning framework for in silico screening of anticancer drugs at the single-cell level.. Natl Sci. Rev..

[141] Lim J., Chin V., Fairfax K., Moutinho C., Suan D., Ji H., Powell J.E. (2023). Transitioning single-cell genomics into the clinic.. Nat. Rev. Genet..

