# Supplementary material for: Single-Cell Transcriptomics: Technical Advances, Applications and Challenges in Cancer Drug Discovery
Source: Curr Cancer Drug Targets. 2025 Jul 30;26(10):1283–97. doi: 10.2174/0115680096372729250715050452 (PMC13681292; doi:10.2174/0115680096372729250715050452)
Supplement: Supplementary file 1 [file CCDT-26-10-1283_SD1.pdf]

Supplementary Material

Single-Cell Transcriptomics: Technical Advances, Applications and Challenges in Cancer Drug Discovery

Yueying Yang[1], Lingyu Meng[1], Teng Zhang[1] and Jianjun Tan[2,*]

[1]*Department of Biomedical Engineering, School of Chemistry and Life Science, Beijing University of Technology, Beijing 100124, China;* [2]*Beijing International Science and Technology Cooperation Base for Intelligent Physiological Measurement and Clinical Transformation, Beijing 100124, China*

Table S1. Key features summary of different Single-cell transcriptomics/Spatial transcriptomics technologies.

Technology Types	Methods	Publication Year	Notes	References
Single-cell transcriptomics	Tang’s method	2009	Micromanipulation	[22]
	STRT-seq	2011	FACS	[40]
	Smart-seq	2012	FACS	[34]
	CEL-seq	2012	Micromanipulation	[38]
	MARS-seq	2014	FACS	[37]
	Drop-seq	2015	Microdroplets	[44]
	InDrop	2015	Microdroplets	[45]
	Seq-Well	2017	Nanowell	[43]
	SCAN-seq	2020	Micromanipulation	[35]
	Smart-seq3xpress	2022	FACS	[33]
	VASA-seq	2022	FACS	[31]
	SCAN-seq2	2023	FACS	[36]
Single-cell multi-omics	G&T-seq	2015	genome , transcriptome	[48]
	DR-seq	2015	genome , transcriptome	[49]
	scTrio-seq	2016	genome, epigenome and transcriptome	[50]
	scM&T-seq	2016	epigenome , transcriptome	[51]
	CITE-seq	2017	surface proteins , transcriptome	[52]
	REAP-seq	2017	surface proteins , transcriptome	[53]
	scDam&T-seq	2019	protein-DNA contacts , transcriptome	[54]
	SNARE-seq	2019	chromatin , transcriptome	[55]
	scCAT-seq	2019	chromatin , transcriptome	[56]
	scNOMeRe-seq	2021	genome-wide chromatin accessibility, DNA methylation and RNA expression	[57]
	snmCAT-seq	2022	methylome, transcriptome and chromatin	[58]
Spatial transcriptomics	tomo-seq	2016	Microdissection-based	[63]

	TIVA	2014	Microdissection-based	[64]
	FISSEQ	2015	Fluorescence imaging (ISS-based)	[65]
	10x Visium	2016	Spatial barcoding	[71]
	NICHE-seq	2017	Microdissection-based	[66]
	Geo-seq	2017	Microdissection-based	[67]
	seqFISH+	2019	Fluorescence imaging (ISH-based)	[68]
	PIC-seq	2020	Microdissection-based	[69]
	STRP-seq	2021	Microdissection-based	[73]
	PIXEL-seq	2021	Spatial barcoding	[70]
	Stereo-seq	2022	Spatial barcoding	[74]
Spatial multi-omics	DbiT-seq	2020	mRNAs , proteins	[129]
	Spatial-CITE-seq	2022	proteins , transcriptome	[130]
	MISAR-seq	2023	ATAC , RNA	[131]
	MiP-seq	2024	DNA , RNA and proteins	[132]
